# Mutational analysis of *Escherichia coli* GreA protein reveals new functional activity independent of antipause and lethal when overexpressed

**DOI:** 10.1038/s41598-020-73069-1

**Published:** 2020-09-30

**Authors:** Llorenç Fernández-Coll, Katarzyna Potrykus, Michael Cashel, Carlos Balsalobre

**Affiliations:** 1 Intramural Research Program, Eunice Kennedy Shriver NICHD, NIH, Bethesda, MD USA; 2 Department of Bacterial Molecular Genetics, Faculty of Biology, University of Gdansk, Gdansk, Poland; 3 Department of Genetics, Microbiology and Statistics, Faculty of Biology, University of Barcelona, Barcelona, Spain

**Keywords:** Bacterial genetics, Bacterial physiology, Bacterial transcription, Microbial genetics

## Abstract

There is a growing appreciation for the diverse regulatory consequences of the family of proteins that bind to the secondary channel of *E. coli* RNA polymerase (RNAP), such as GreA, GreB or DksA. Similar binding sites could suggest a competition between them. GreA is characterised to rescue stalled RNAP complexes due to its antipause activity, but also it is involved in transcription fidelity and proofreading. Here, overexpression of GreA is noted to be lethal independent of its antipause activity. A library of random GreA variants has been used to isolate lethality suppressors to assess important residues for GreA functionality and its interaction with the RNA polymerase. Some mutant defects are inferred to be associated with altered binding competition with DksA, while other variants seem to have antipause activity defects that cannot reverse a GreA-sensitive pause site in a *fliC::lacZ* reporter system. Surprisingly, apparent binding and cleavage defects are found scattered throughout both the coiled-coil and globular domains. Thus, the coiled-coil of GreA is not just a measuring stick ensuring placement of acidic residues precisely at the catalytic centre but also seems to have binding functions. These lethality suppressor mutants may provide valuable tools for future structural and functional studies.

## Introduction

Transcription in bacteria is catalysed by the RNA polymerase (RNAP), a multisubunit enzyme with a core formed by 5 subunits, α_2_ββ′ω. Subunits β and β′ form the catalytic centre, where two Mg^2+^ ions are crucial for the RNA synthesis by coordinating structures from both subunits^1,2^. The three-dimensional structure of the RNAP defines two spaces that play a relevant role during transcription, the primary and the secondary channel. The primary channel is occupied by the DNA and that is where the nascent RNA is synthesized. The secondary channel is an entryway for nucleotides and also for several transcription factors interacting with RNAP including the secondary messenger ppGpp and several proteins ^3–8^. Several small proteins interact with the secondary channel of *E. coli* RNAP and share similar structures: DksA^2^, GreA, GreB^9^ and Rnk^7^. The global regulator ppGpp often acts with the help of DksA by modulating expression of a large number of genes^10–16^. GreA and GreB, interact within the secondary channel of RNAP to reverse transcription pausing during elongation by cleaving backtracked transcripts and restore transcription^17^. GreB cleaves longer backtracked nascent RNA chains than GreA^9^. Δ*greA* mutants exhibit several phenotypes, including sensitivity to salt and to divalent metals^18^. In contrast, Δ*greB* deletions are phenotypically normal. Transcriptomic studies show multiple adjustments of gene expression by elevated levels of GreA in the absence or presence of DksA, suggesting a functional interplay among the ppGpp/DksA regulatory components and the Gre factors^19^. Several reports show that these factors are important for DNA stability by avoiding collisions between replication and transcription machinery or by enhancing transcription fidelity and avoiding backtracking^20–22^. However, GreA inhibits double strand break repair and its absence promotes *recA* facilitation of *recBCD* repair^23^. Lastly, it has been suggested that the Rnk protein, by interacting with the secondary channel of RNAP, might inhibit GreA functions under certain conditions^7^.

Competition has been proposed to occur at the level of binding among the different proteins that interact with the secondary channel^19,24,25^. Consequently, alterations in the amount of any secondary channel protein and/or changes in their affinities for the secondary channel may affect gene expression. Data support this hypothetical model. Divergent effects between DksA and ppGpp deficient strains have been attributed to the presence of GreA^14,26,27^. Complex cross-talk has been inferred among several of the factors that interact with the secondary channel of RNAP^19^. Conformational changes in the structure of DksA might affect its affinity for RNAP^28–30^ and therefore alter the hypothetical competition.

In *E. coli*, GreA is a 158 aa protein with a molecular mass of 17.5 kDa. Two distinct domains can be defined, an N-terminal coiled-coil domain formed by two antiparallel α-helices linked by a turn, and a C-terminal globular domain that contains one β-barrel and one α-helix; the two domains are linked by a flexible linker^31,32^. To resolve backtracked complexes during transcription, the coiled-coil domain of the Gre factors enters through the secondary channel of RNAP and activates an intrinsic endoribonuclease activity that cleaves backtracked nascent RNA to restore the proper positioning of the 3′-end of the transcript within the active centre so that elongation may resume. The GreA acidic residues D41 and E44 at the tip of the coiled-coil participate in an essential interaction with the Mg^2+^ ion in the RNAP catalytic center^17,33,34^. The globular domain of GreA remains outside of the secondary channel and plays a role in promoting its binding to RNAP^35^. The globular domains of the Gre factors and DksA bind to the β′ rim helices of the secondary channel (V673-E685)^17,36–38^.

In this report an in vivo approach has been used to identify residues of GreA important for its functionality and ability to bind RNAP. Induced overexpression of GreA is found here to lead a severe inhibition of growth and loss of viability, which seems to be independent of GreA’s antipause activity. This feature has been exploited to isolate a library of random missense *greA* mutants that suppress lethality. In vivo assays with a *fliC::lacZ* reporter construct have been designed to distinguish between residues involved in the binding affinity for RNAP from those involved in the cleavage inducing activity of GreA.

## Results

### Overexpression of GreA has a deleterious effect over bacterial growth, enhanced by the absence of DksA

**Figure 1:**
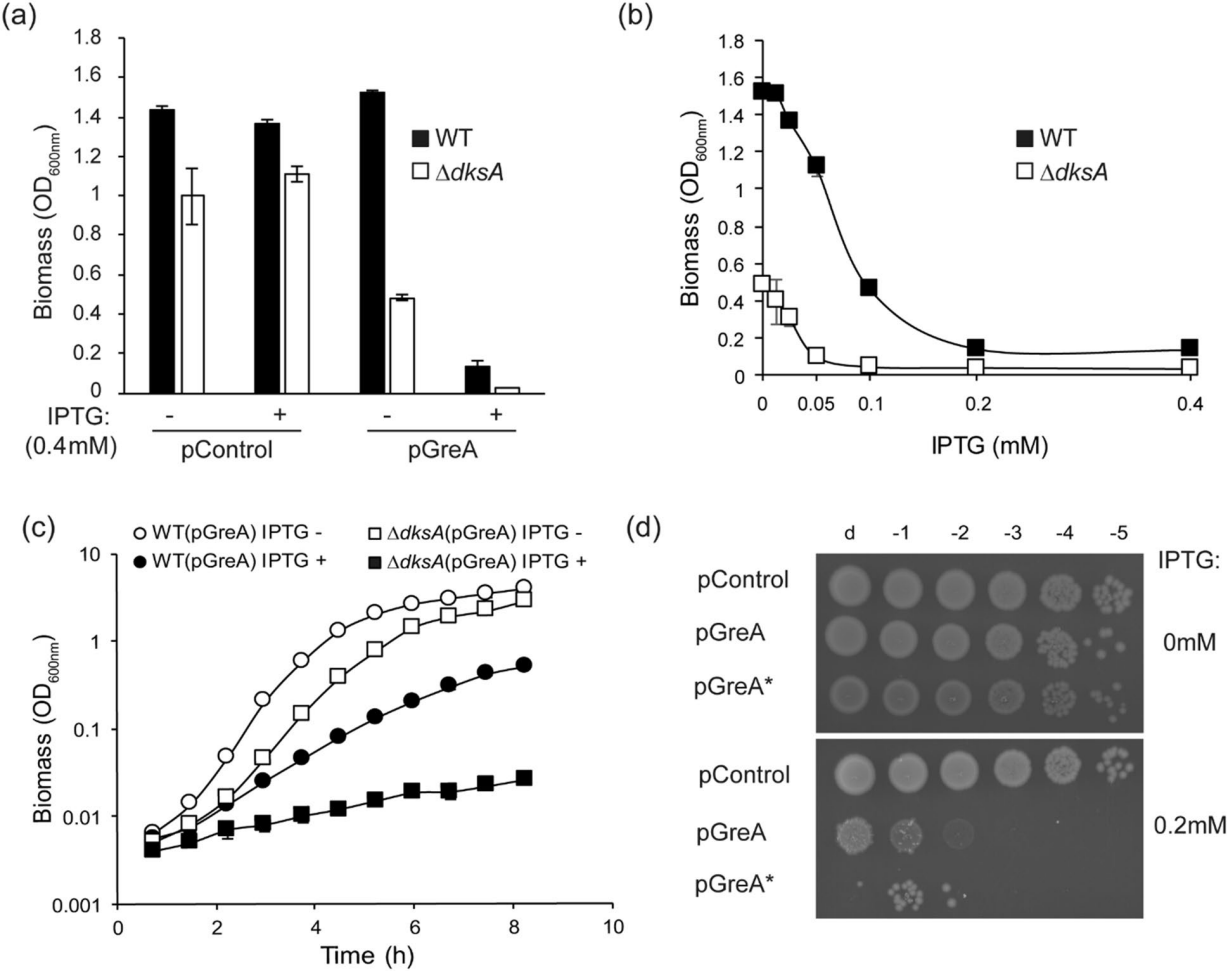
Effects of GreA overexpression on growth. (**a**) Strains MG1655 (WT) and TE8114 (Δ*dksA*) were transformed with pTrc99A (pControl) and pDNL278 (pGreA) and grown for 12 h in LB at 30 °C in presence or absence of IPTG (0.4 mM) then OD_600nm_ measured. (**b**) Effect of increasing IPTG induction of the same strains, grown as in panel (**a**). (**c**) Strains MG1655 and TE8114 transformed with pGreA and as in panel (**a**) but at 37 °C, and with or without 0.2 mM IPTG. (**d**) MG1655 (WT) harbouring plasmid pTrc99a (pControl), pDNL278 (pGreA) and pHM1701 (pGreA*) grown in LB to similar densities and then serially diluted, with 2 μl of each dilution applied to LB plates containing 0.2 mM IPTG or without inducer. Error bars represent SD from 2 independent cultures.

To identify pivotal amino acid residues for GreA activity and its interaction with RNAP, the GreA protein was overexpressed using pDNL278, a multicopy plasmid carrying the *greA* gene under the control of the IPTG inducible promoter Ptac^39^. Effects on growth were monitored in Wt and Δ*dksA* (TE8114) strains. Cultures of the strains carrying either plasmid pDNL278 (pGreA) or pTrc99a (pControl) were grown in LB at 30 °C for 12 h with or without 0.4 mM IPTG. These growth conditions let the Wt strain to reach stationary phase and to allow optimal comparisons within the different strains. Bacterial growth was monitored by determination of OD_600nm_ of the cultures (Fig. 1a). No important differences were observed between the Wt and Δ*dksA* strains carrying the vector control in the presence or absence of IPTG (Fig. 1a). In contrast, IPTG-inducible overexpression of GreA produces a negative effect in both strains, Wt and Δ*dksA*. Moreover, Δ*dksA* cells seem to be more sensitive to GreA overexpression than Wt, because even the presence of the uninduced pGreA plasmid produces a reduction of growth yield about fourfold (Fig. 1a). It is important to note that in Wt cells, the amount of DksA is 2.5-fold higher than the amount of GreA, and both are in excess when compared to RNAP^40^. Moreover, the uninduced pGreA plasmid will increase the levels of GreA up to sixfold (Supplementary Fig. S1a). Therefore, in Δ*dksA* cells, GreA expressed from the uninduced plasmid will freely bind to the RNAP, producing a negative effect on bacterial growth.

Effects of IPTG titration (0, 0.0125, 0.025, 0.05, 0.1, 0.2 and 0.4 mM) on growth are shown in Fig. 1b. As IPTG increases, inhibition of growth occurs in both strains. When *dksA* is deleted, IPTG sensitivity is markedly enhanced. Considering that in *lacY* + strains, such as MG1655, the effect of IPTG can be bistable instead of linear, a similar experiment was performed with a *lacY* mutant strain (Supplementary Fig. 1b). A linear decrease in bacterial growth up to a concentration of 0.2 mM IPTG was observed for both strains.

The effects of GreA induction on exponential growth rates in LB media confirm that the *dksA* deletion renders GreA overexpression more toxic when induced with 0.2 mM IPTG (Fig. 1c). The generation time of the Wt strain increases from approximately 25 min to 50 min, whereas the generation time of the *dksA* deletion strain increased from 30 min to about 150 min when GreA is induced. We infer that the lack of DksA binding the secondary channel of RNAP is likely to promote GreA access, which leads to amplified GreA-dependent growth inhibition.

### Antipause activity is not required for the deleterious effect of GreA overexpression

In order to determine if the antipause activity is required for the deleterious effect of GreA overexpression, a GreA allele carrying a mutation in the acidic residues (D41 and E44) responsible for activating cleavage resulting in antipause activity was overexpressed in Wt background. MG1655 carrying either pControl, pGreA or pGreA D41A E44Y were grown to similar densities in absence of inducer (IPTG), then diluted and spotted on LB agar plates −/+ 0.2 mM IPTG (Fig. 1d). As expected, in absence of IPTG no differences in the bacterial count were observed, but when either GreA or GreA D41A E44Y were overexpressed in the presence of IPTG, a negative effect was observed (Fig. 1d).

The toxicity observed does not seem to reflect a more general protein misfolding problem that can accompany overexpression of many proteins. Similar experiments were performed overexpressing separately the GreA coiled-coil domain (NTD) or the globular domain (CTD). Neither shows such a lethal effect (Supplementary Fig. S2a). While NTD has been described to be responsible for the antipause activity, both the NTD and CTD domains seem required for the specific binding of GreA to the RNAP^41^.

### Selection of *greA* intragenic missense suppressors that restore growth

The lethal effects of GreA overexpression can be exploited to isolate *greA* mutants that suppress lethality. Considering that GreA interacts with RNAP it will be assumed that any suppressor mutants either weaken their binding affinity to RNAP or affect the functionality of GreA. It is also possible that a single mutation might cause partial loss of both functions.

**Figure 2:**
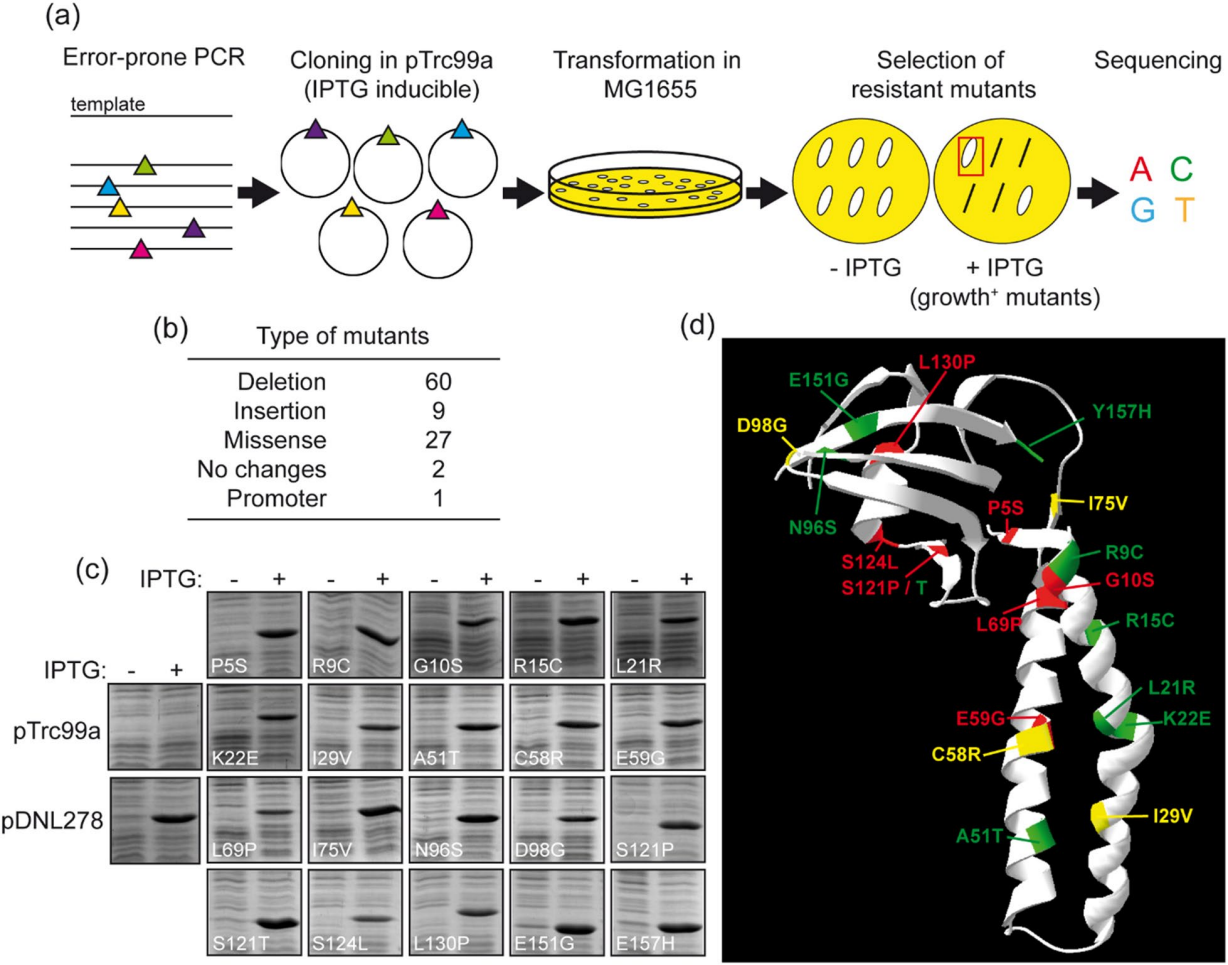
Mutagenesis of GreA by Error-Prone PCR. (**a**) Scheme of the random mutagenesis experiment. The colored triangles represent nucleotide mutations. The conditions of no-induction (–) and induction of *greA* overexpression are indicated as (−) and (+), respectively. (**b**) Characterisation of the different types of mutations obtained after selecting resistant mutants. The number of mutants that carry deletions, insertions, missense mutations, mutations in the promoter or that do not contain any mutation either in the *greA* coding sequence or in the Ptac promoter region (no changes) is indicated. (**c**) GreA production in MG1655 from plasmids pTrc99a (pControl), pDNL278 (pGreA) and the set of 20 pTrc-greA mutants. Strains were grown in LB at 37 °C to an OD_600nm_ of 0.1, then induced or not induced with 0.2 mM IPTG for 3 h. Whole cell extracts were subjected to 12.5% SDS-PAGE and stained with Coomassie brilliant blue. (**d**) Distribution of the different mutations on the 3D structure of GreA. Mutant classes are colour coded: Class I red, Class II green and Class III yellow.

The experimental strategy to generate a library of *greA* random mutants is depicted in Fig. 2a. First, the *greA* gene from MG1655 strain was amplified by error-prone PCR and the resulting PCR fragments, presumably carrying random mutations, were subsequently cloned into pTrc99a and transformed into MG1655. The resulting clones were streaked on LB plates with or without IPTG (0.2 mM) to select clones able to grow under GreA overexpression conditions. Finally, the *greA* alleles cloned in the pTrc99a vector of the selected clones (growth^+^) were sequenced.

Up to 484 clones were obtained and the ability to grow on LB plates supplemented with IPTG was scored. From those, 99 (20.4% of the clones) were able to grow in the presence of IPTG (growth^+^ mutants), and all of these *greA* alleles were sequenced. Considering that GreA is not essential under the growth conditions assessed, it is easier for the cell to delete it rather than selecting point mutations that would weaken their function or ability to bind. Then, as shown in Fig. 2b, the vast majority of the growth^+^ clones carry deletion mutations (60 of the 99), whereas only 27 growth^+^ clones contain missense mutations causing amino acid changes in the sequence. Substitutions in 20 different amino acids of the GreA protein, 12.6% of its sequence, were detected. Accordingly, 7 out of 27 missense mutants (P5S, G10S, L69P, I75V, N96S, S124L and L130P) were repeated mutations that were not considered for the forthcoming study. The ability of overproduction of the different plasmids was corroborated for the 20 GreA mutants (Fig. 2c).

### Mutant characterisation

The mutations are localized in both the coiled-coil and globular GreA structural domains (domains defined as in Stebbins et al.^32^). Twelve GreA variants carry mutations within the coiled-coil N terminal domain (residues 5–76) and eight in the C-terminal globular domain (residues 91–158), Fig. 2d. No mutants were found that altered the two acidic residues (D41 and E44), consistent with the fact that GreA D41A E44Y is also lethal when overexpressed (Fig. 1d). The ability to form colonies when overexpressed in Wt and Δ*dksA* strains of the different mutants was scored on LB plates with 0.2 mM IPTG (Table 1). Their pattern of growth allowed us to sort the *greA* alleles into three classes. Class I mutants grow with or without DksA, suggesting a loss of the lethal activity, unaltered by the absence of competition with DksA for binding. This can be due to a loss of the activity per se or due severe altered binding of GreA to RNAP. Class II mutants grow in the Wt host but not in the Δ*dksA* strain. We might infer that this class arises primarily because of defects in their ability to compete against DksA for binding to polymerase rather than defects associated with the lethal activity. Class III mutants have an intermediate growth phenotype since they grow poorly when induced in the Δ*dksA* host, but they show normal colony size without induction. Localization of the three classes of the pTrc-GreA^Mut^ set are colour coded in Fig. 2d: Class I red, Class II green and Class III yellow. To further characterise the mutants, their antipause activity and ability to bind the RNAP were tested.

**Table 1:** Summary of GreA mutant characteristics. Effect of overexpression of the different *greA* alleles on MG1655 (Wt) and TE8114 (dksA) strains on LB plates supplemented with 0.2 mM IPTG (described in the text). The antipause activity and ability to bind to the RNAP is indicated for each mutant.

	pControl	pGreA	Frameshift	P5S	R9C	G10S	R15C	L21R	K22E	I29V	A51T	C58R	E59G	L69P	I75V	N96S	D98G	S121P	S121T	S124L	L130P^e^	E151G	Y157H
**Growth**^a^																							
Wt	++	−	++	+	+	++	+	+	++	++	+	++	+	++	++	++	++	++	++	++	++	+	+
*dksA*	++	−	++	+	−	++	−	−	−	+	−	+	+	++	+	−	+	++	−	++	++	−	−
	Class^b^			I	II	I	II	II	II	III	II	III	I	I	III	II	III	I	II	I	I	II	II
% Actv.^c^	7	100	11	34	95	60	59	59	44	73	85	39	29	27	68	90	45	22	35	9	8	107	96
Binding^d^				RA	NC	B	NC	NC	NC	B	NC	RA	B	RA	B	NC	B	RA	NC	B	–	NC	NC
% Sol.^f^	–	95	–	89	87	93	86	98	96	72	100	99	96	94	93	93	72	88	83	94	–	100	91

^a^Ability of the different GreA mutants to grow when overexpressed in different backgrounds: Wt or Δ*dksA*. Scored as ++ when colony size resembles vector control (resistant), + when colony size is smaller than vector control (resistant-intermediate) and – when no growth is detected.

^b^Class of mutants as described in the text.

^c^Antipause activity: percentage of suppression of *fliC* expression in strain PRG18, normalized to the induction of pGreA (set as 100%), as shown in Fig. 3

^d^Binding to the RNAP as estimated by in vivo assay shown in Fig. 4**.** The mutants were scored as: Binding not affected (B), Reduced Affinity (RA), or Not able to Compete with DksA (NC). When underlined, binding ability was also tested by a competition assay (Fig. 5).

^e^Protein L130P is unstable, therefore, no further study was performed.

^f^Solubility measured based on Supplementary Fig. S4.

### Measurement of antipause activity of the different GreA variants using a *fliC*::*lacZ* reporter assay

A β-galactosidase reporter assay has been developed which measures GreA-mediated reversal of arrests during transcription elongation of the *fliC* gene, coding the major flagella subunit. As described by Åberg et al.^14^, the expression from a *fliC::lacZ* distal fusion (+ 1210) prominently increases in a Δ*dksA* strain as compared to Wt, whereas in a Δ*dksA* Δ*greA* strain the expression returns to Wt levels (Supplementary Fig. S3a). Remarkably, the GreA-mediated induction of *fliC* expression does not occur during transcription initiation phase since it was not detected with a proximal fusion (+ 70)^14^. Accordingly, in the PRG18 strain, a Δ*dksA* Δ*greA* derivative carrying the *fliC*_+1210_::*lacZ* fusion, the *fliC* expression is very low, whereas it is greatly induced when *greA* is expressed in trans, by introducing the plasmid pGreA. Furthermore, the GreA D41A E44Y variant, described to be impaired to suppress arrested complexes, did not induce *fliC* transcription (Supplementary Fig. S3b). In this setting, since the chromosomal *greA* and *dksA* are deleted, the competition between secondary channel factors doesn’t have to be accounted for, allowing to score the GreA variants for their effectiveness in rescuing stalled RNAP complexes.

**Figure 3:**
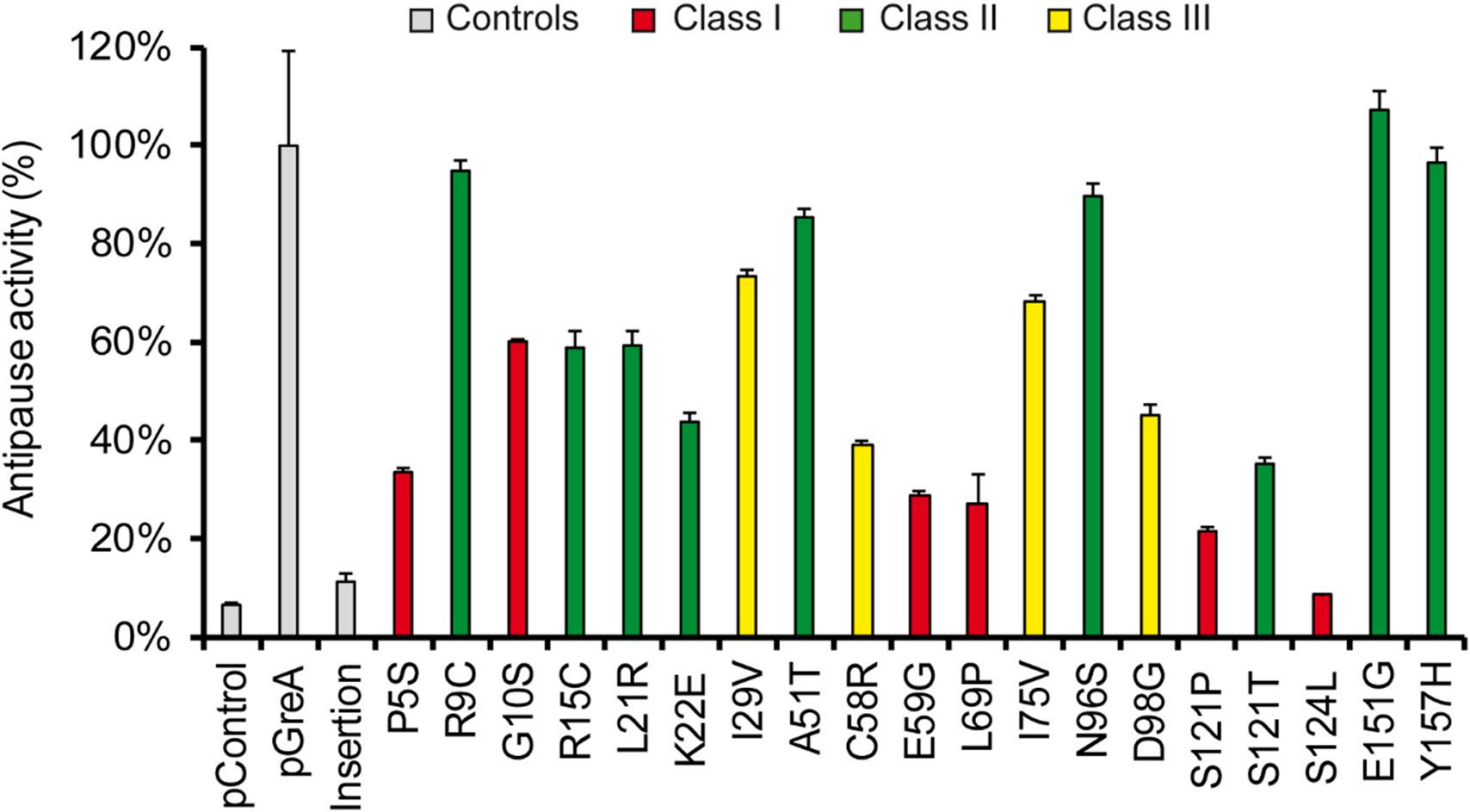
*fliC*::*lacZ* expression monitors in vivo GreA reversal of transcription elongation arrest in Δ*dksA* Δ*greA* host cells carrying pGreA derived plasmids. *fliC*-*lacZ* β-gal activities are made in the PRG18 strain (Δ*dksA* Δ*greA fliC*::*lacZ* distal) bearing plasmids pTrc99a (pControl), pDNL278 (pGreA) or each of the 20 different pTrc-GreA^Mut^. Cultures were grown in LB at 37 °C up to an OD_600nm_ 1.5. The β-galactosidase activities shown are normalized to pGreA, set as 100% of antipause activity. Error bars represent SD from 2 biological replicates and 3 technical replicates. Mutant classes are colour coded as in Fig. 2c: Class I red, Class II green, Class III yellow.

The PRG18 strain (*fliC::lacZ*_+1210_, Δ*dksA* Δ*greA*) was transformed with the pTrc-GreA^Mut^ set as well as the three controls: the pTrc vector, the pGreA positive control and a negative control with an early frameshift mutation producing a stop codon. Cultures of the transformants were grown in LB and the *fliC::lacZ*_+1210_ expression was monitored by determination of the β-gal activity. Values were expressed as percentage of the antipause activity, normalizing to *fliC* expression measured in the strain carrying the Wt GreA protein as 100% of antipause effect (Fig. 3 and Table 1). The basal expression of pGreA positive control gave a 15-fold increase (compared to pControl) in the Lac activity and consequently, no IPTG was used in the cultures to monitor *fliC* transcriptional expression. The pTrc-GreA^Mut^ set gave a wide range of *fliC* expression levels, indicating variable abilities to rescue paused backtracked transcription complexes. Interestingly, all Class I mutants showed a reduction in their ability to rescue paused RNAP, with their activity being lower than 35% of the activity displayed by pGreA (except for G10S, whose activity level reached 60% of Wt pGreA). The lowest activities were shown by distal α-helix globular domain mutant alleles: S124L (9%) and L130P (8%). Class II mutants were projected to be primarily affected in their competition abilities with DksA. All nine Class II alleles showed GreA antipause activities that were decreased when compared to Wt GreA, but that were at least greater than 40% activity of Wt pGreA, i.e. more active than Class I mutants (except for G10S). Three of the Class II alleles (R9C, E151G and Y157H) gave *fliC* expression levels that approximate or even exceed those of pGreA, as if they display no antipause defect at all. The Class III mutants ranked somewhere between Class I and Class II mutants, on average.

The soluble GreA basal levels were measured for the different mutant alleles in the PRG18 strain (Supplementary Fig. S4a and S5). The mutant L130P has undetectable protein, and it was not used for further studies. The mutants A51T, N96 and D98G, display a noticeable protein reduction that could explain the effects observed in Fig. 3. Although S124L shows a twofold decrease in the basal levels, it does not account for the loss of 90% of the activity seen in Fig. 3. The rest of the mutants show similar amounts of protein as pDNL278.

### Competition of GreA variants for binding to the RNA polymerase

**Figure 4:**
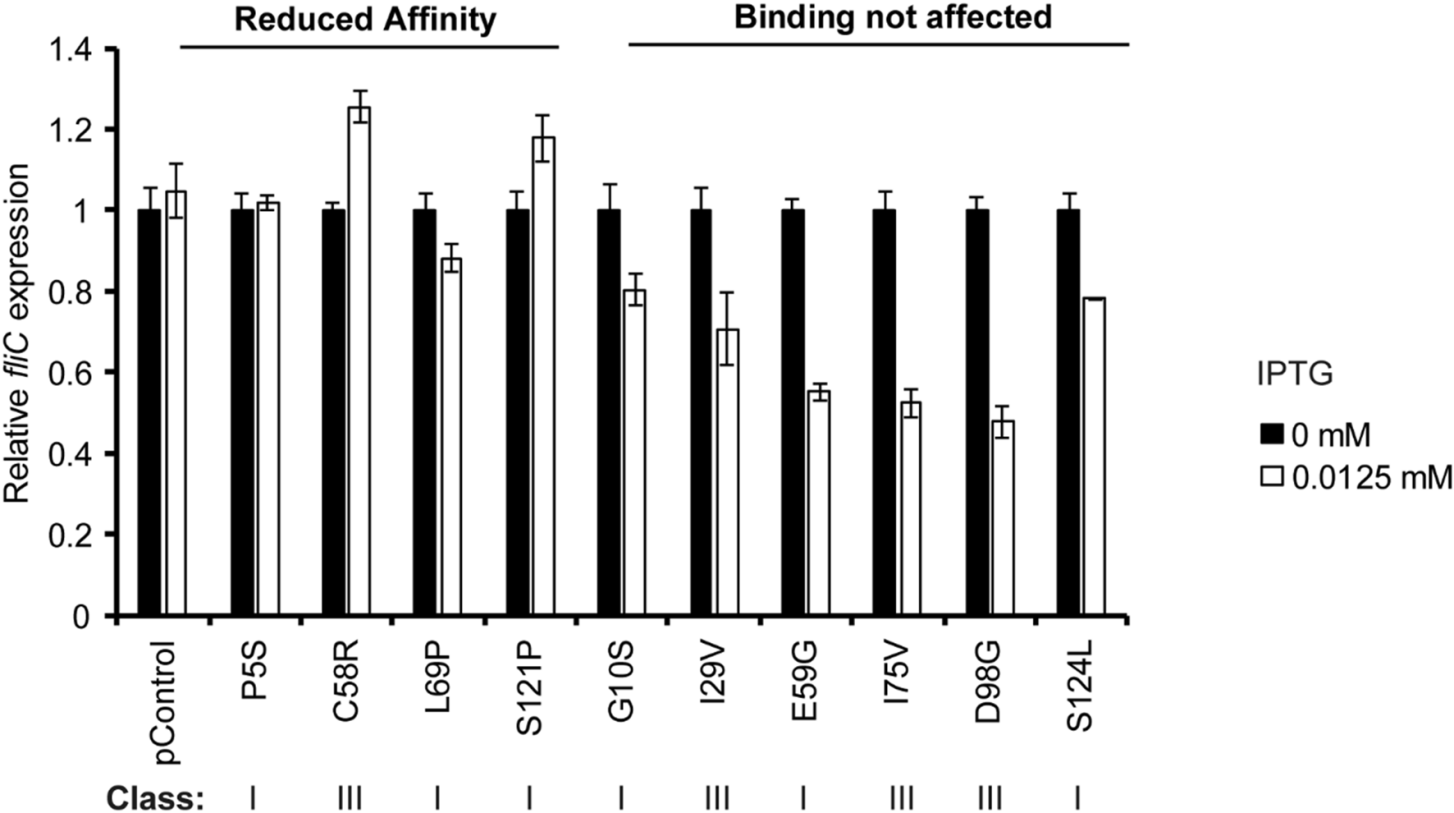
*fliC*::*lacZ* expression in the presence of weakly induced GreA variants in the *greA* + Δ*dksA* host cells. PRG17 (Δ*dksA fliC*::*lacZ* distal) strains carrying the plasmid pTrc99a (pControl) or the indicated Class I and III pTrc-GreA^Mut^ alleles were grown in LB with or without induction by 0.0125 mM IPTG. Cultures were grown at 37 °C in LB and assayed for β-galactosidase activity at OD_600nm_ of 1.5 and their activities were normalized to activities of uninduced cells. Error bars represent SD from 2 biological replicates and 3 technical replicates. The Class of each mutant is indicated below.

Next, the RNAP binding ability of the GreA variants’ was monitored using an in vivo assay based on the *fliC*::*lacZ* reporter assay. As shown in Supplementary Fig. S3a, the expression of *fliC* increases in a *dksA* mutant strain, presumably due to enhanced binding of GreA to the secondary channel of RNAP, since the increase is strictly dependent on the presence of GreA. In this scenario, overexpression of a GreA variant with unaltered affinity for RNAP might compete with the chromosomal GreA for binding to the RNAP. If the overexpressed GreA variant is not functional, but is binding to RNAP with similar affinity, a decrease in *fliC* expression is expected. On the other hand, if the overexpressed GreA variant has a reduced affinity to RNAP, it would not compete with the chromosomal GreA and the expression of *fliC* should not vary. Therefore, strain PRG17 (*fliC::lacZ*_+1210_, Δ*dksA*) was transformed with the Class I and III alleles from the pTrc-GreA^Mut^ set of plasmids, induced with low levels (0.0125 mM) of IPTG and again assayed for β-galactosidase reporter activity. These low levels of IPTG reduced bacterial growth up to 20% in a Δ*dksA* strain containing pGreA (Fig. 1b) and were used to minimize the deleterious effects on growth of overexpressing class III mutants in a Δ*dksA* strain. The class I mutant L130P was not involved in this study due to the low expression detected (see above). The results for six Class I mutants and four Class III mutants are shown normalized to uninduced controls in Fig. 4. As mentioned above, Class II mutants were not assessed since when overexpressed they do not support growth in the absence of DksA.

Overexpression of the G10S, I29V, E59G, I75V, D98G and S124L variants decreases expression of *fliC*. This suggests that these GreA variants, although showing a reduced antipause activity, can bind to the RNAP and displace the chromosomally encoded GreA^Wt^. However, overexpression of the P5S, C58R, L69P and S121P variants has no effects on the expression of *fliC* as if they are unable to bind to the RNAP. Three of these alleles are in Class I (P5S, L69P, S121P) and one is in Class III (C58R).

**Figure 5:**
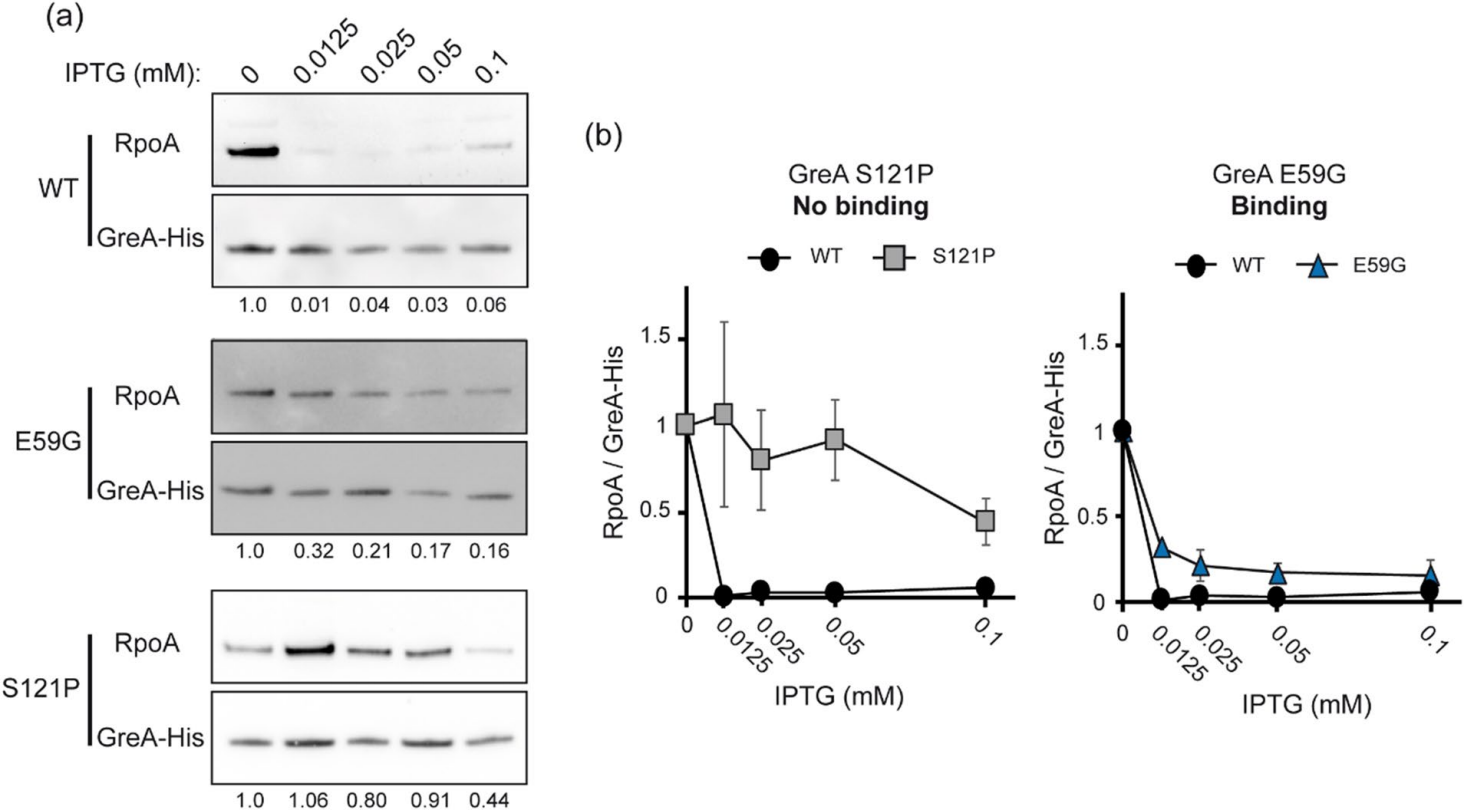
GreA binding to the RNA polymerase. Validation of the binding activity conclusions from the *fliC*::*lacZ* activity tests (Fig. 4) by co-purification of RNAP RpoA subunit with affinity tagged GreA-His. (**a**) Western blots with anti-RpoA and anti-GreA-His were performed using the CF14758 strain (Δ*greA* Δ*greB*) containing pBb-GreA-his and pDNL278 (pGreA^WT^), pTrc-GreA^S121P^ or pTrc-GreA^E59G^. Control Western blots showing protein abundance detected by Anti-RpoA or Anti-GreA-His in extracts before copurification are shown in Supplementary Fig. S6. Full western blots are shown in Supplementary Fig. S7. (**b**) RpoA abundance is normalized to His-tagged GreA abundance without IPTG. Error bars represent SD from 2 biological and technical replicates.

Pull down assays were performed to verify the binding defects that were deduced from the in vivo competition assays. Binding of two GreA variants to RNAP, as compared to GreA^Wt^ was determined. The two GreA variants studied show different behaviour in the in vivo competition assays: E59G displays normal binding while S121P show a reduced ability to bind. For this assay, as described in the Materials and Methods section, the pBb-greA-His plasmid was constructed expressing a Wt GreA protein with a C-terminal His-tag from an anhydrotetracycline (anTc) inducible promoter. This plasmid was transformed into CF14758 strain (Δ*greA* Δ*greB*) carrying either pDNL278 (pGreA), pTrc-*greA* S121P or pTrc-*greA* E59G. These strains were grown in LB to an OD_600_ of 0.1, then 0.2 µM of anTc was added. After one hour of induction of GreA-His, expression of competitor GreA protein (GreA Wt, GreA S121P or GreA E59G) was induced with increasing amounts of IPTG for an additional 30 min. RNAP bound to affinity purified GreA-his from the whole cell extracts was estimated by Western blots as ratios of RNAP α-subunit (RpoA) abundance normalized to GreA-his protein. If competition for binding occurs, a decrease in the level of RNAP co-purifying with GreA-His is expected. If binding is defective and competition does not occur, then the amount of RNAP that co-purifies with GreA-His should remain high. Figure 5 shows that, as expected from binding competition, when GreA Wt is overexpressed from pGreA, a dramatic decrease occurs in the amount of RpoA that co-purifies. A similar behaviour was observed with GreA E59G that was found by the in vivo assay to be able to bind to RNAP (Fig. 4). In contrast, the amount of RpoA co-purified with GreA-His was marginally altered when the deduced binding defective GreA S121P allele was overexpressed. Control Western blots were performed to verify expression predictions of His-tagged-GreA protein, untagged-GreA-variants and RpoA induced with anTc and IPTG in whole cell extracts (Supplementary Fig. S6). All together, the results can be taken to validate the findings of the *fliC*-based in vivo reporter system for measuring the secondary channel binding of mutant GreA proteins.

## Discussion

Transcription factor GreA binds to the secondary channel of the RNAP to rescue backtracked polymerases^17,42^ and is an important transcriptional proof-reading factor^43^, although its overexpression is shown here to be lethal for *E. coli* (Fig. 1). Although the mechanism of such lethality is unknown, it seems to be independent of the GreA antipause activity (Fig. 1d). Despite abundant information on GreA, there are still some surprises in our understanding of its function. This is often addressed using random mutant libraries in search for responses to unexplained behaviour, without making assumptions as to the nature of functions affected. Random mutant suppressor libraries have been used to study the interactions of DksA with RNAP and ppGpp^44^. The library generated here allowed us to determine residues within the GreA protein important for this lethal activity, but also paying special attention to any alteration in the known antipause function or binding to the RNAP.

As mentioned earlier, the protein levels of DksA (~ 137 fmoles/μg of total protein) are up to 2.5 times higher than the levels of GreA (~ 53 fmol/μg of total protein)^40^. Although the levels of RNAP seem to vary depending on the growth media (~ 46 fmol/μg of total protein in rich media compared to ~ 14 fmol/μg of total protein in minimal media)^45^, the levels of DksA and GreA seem to be the same or higher than the amount of RNAP. The Kd of 0.1 µM of DksA for RNAP is lower than that of GreA (0.8 µM)^40^, suggesting that DksA has a higher affinity for the RNAP than GreA under the conditions tested. However, competition studies^26^ show that GreA can displace already bound DksA and compete for the RNAP. But it is more complicated, since DksA from *E. coli* and *Salmonella*^28–30^ undergo conformational changes that modify their affinity for the RNAP.

Gfh1 from *Thermus aquaticus* is structurally similar to GreA and has a highly flexible interdomain linker that by responding to changes in pH varies its orientation producing active and inactive conformations^46–48^. Interestingly one of the mutants obtained in this study (I75V) is located at the edge between the coiled-coil domain and the flexible linker. Although the change from Ile to Val does not involve a change in hydrophobicity, 34% of GreA’s antipause activity is lost when compared to Wt (Table 1). The structural proximity of I75 with P5 and F88 might allow hydrophobic interactions between the globular and coiled-coil domains; I75V and P5S mutants found here concern two of these three residues.

Typically, it has been described that the binding activity of GreA resides in the globular domain^17,35,49^, and the cleavage activity resides in the residues D41 and E44^17,33^. Then, one might think that the coiled-coil is basically a spacer to introduce D41 and E44 into the secondary channel and assure proper contact with the catalytic centre. Some mutants located in the coiled-coil domain (P5S, C58R and L69P) show a reduced ability to bind RNAP, suggesting that the coiled-coil seems to contact RNAP ensuring binding of GreA. At the same time, mutations in the globular domain, such as S124L, appear to display strongly defective antipause activity of GreA and yet preserve an ability to bind to RNAP. It is worth to note that in some cases the effects on transcriptional arrest ultimately rely on precise placement of the two acidic residues at the tip of the coiled-coil domain, for example: E59G. The E59 amino acid interacts through a salt bridge with R26^32^ and change from E to G (E59G) should prevent this interaction, affecting the descending helix and changing the orientation of the D41 and E44 residues. The outcome is a reduction in the antipause activity up to 75% (Table 1).

A transcriptomic study^50^ determined changes in gene expression using similar GreA overexpression conditions: same plasmid as used in our study (pDNL278) and cells were grown in LB with 0.05 mM of IPTG at 30 °C (bacterial growth is reduced up to 25% in Wt strain under these conditions, as shown in Fig. 1b). Under those conditions, gene expression of several chaperones and heat shock proteins decreases significantly, as well as that of several genes involved in central metabolism, TCA cycle and cellular division. Most of the genes upregulated by GreA overexpression are ribosomal proteins or involved in respiration^50^. Although most of the transcriptomic effects of GreA have been associated with GreA’s antipause activity, some of them seem to be produced independently of it^19^. It should be also noted that although GreA has been reported to impair DNA double-strand breaks repair^23^, it does require its antipause activity to interfere with repair and, as mentioned above, the lethal activity associated with GreA is independent of its antipause activity (Fig. 1d).

Another possibility is more mechanistic. Structural studies^51^ of backtracked RNAP binding GreB show that GreB needs to be released from the secondary channel to allow transcription to resume. After cleavage of the backtracked RNA, GreB is expelled from the secondary channel and the SI3 domain of the RNAP contacts the rim helices, closing the secondary channel. When GreB is in high concentrations, it can maintain a weak contact with the RNAP after cleavage but outside the secondary channel^51^. One could imagine similar contacts happened with GreA. Therefore, overexpression of GreA could force its binding to the RNAP, which could interfere with the ability of RNAP to transcribe. Other possible effects might include secondary channel blockage by GreA, possibly by binding outside the channel as shown for GreB^51^, and limiting either access to NTP substrates or the binding of other transcription factors to RNAP. As said before, toxicity is lessened when DksA is present and growth toxicity is enhanced when *dksA* is deleted, suggesting that toxicity is due to an excess of GreA outcompeting DksA binding. Our view is that it is simply not possible at this time to propose a precise mechanism of how GreA overexpression results in growth inhibition but the occurrence of mutants in one domain that affects functions thought to be associated with the other domain provides examples that are worth pursuing in the future.

Competition between factors that interact with the secondary channel add another layer of complexity to regulation of gene expression. Although several studies explored the effects of presence or absence of these factors, understanding the mechanisms involved in keeping a balanced equilibrium between binding partners is also required. The amplification of the negative effect of GreA overexpression on *E. coli* growth in the absence of DksA emphasize how important it is that a correct equilibrium exists between the different factors that interact with the RNAP secondary channel. Changes on this equilibrium seem to have severe consequences for the transcriptional apparatus and consequently for the physiology of the cell.

## Methods

### Media and growth conditions

Strains and plasmids used in this study are listed in Table 2. Routinely, cultures were grown in LB broth at 37 °C, unless another temperature (30 °C) is indicated. Minimal medium M9 plates were prepared as previously described^19^. When required, the following antibiotics were added at the indicated concentrations: 50 μg/ml ampicillin (Ap), 12.5 μg/ml tetracycline (Tc), 20 μg/ml chloramphenicol (Cm), 25 μg/ml kanamycin (Km) and 25 μg/ml spectinomycin (Spec).

**Table 2:** Bacterial strains and plasmids used in this study.

Name	Properties	Origin
**Strains**		
MG1655	F-, *ilvG*, *rph*1	^55^
GM2163	F-, λ- *araC*14 *leuB*6 *fhuA*31 *lacY*1 *tsx*-78 *glnX*44 *galK*2 *galT*22 *hisG*4 *rpsL*136(str-r) *xylA*5 *mtl*-1 *thiE*1 *dam*-13::Tn9 *dcm*-6 *hsdR*2 *mcrA- mcrB*-	Cashel’s lab collection
TE8114	MG1655 *dksA::*Tc^R^	^15^
AAG101	MG1655 *dksA::Tc greA::Cm*	^14^
AAG93	MG1655 ∆*relA* ∆*spoT* (ppGpp^0^)	^56^
CF11663	MG1655 greB::*Km*	^14^
CF14758	MG1655 Δ*greA* Δ*greB*	^19^
AAG1	MG1655 ∆*lacZ*	^57^
PRG16	AAG1 *fliC::lacZ* (+ 1210)	^14^
PRG17	AAG1 *fliC::lacZ* (+ 1210) *dksA*::Tc^R^	^14^
PRG18	AAG1 *fliC::lacZ* (+ 1210) *dksA*::Tc^R^ *greA*::Cm^R^	^14^
CAG68251	BW25113 *rpoBC*::Cm *rpoB* (ΔSI1)	^37^
LFC1250	MG1655 *rpoBC*::Cm *rpoB* (ΔSI1) (P1 transduction)	This study
**Plasmids**		
pTrc99a	colE1 origin, *lacI*^q^, P*trc* expression vector, Ap^R^	^58^
pDNL278	*lacI*^q^, *greA* under control of P*trc* on pTrc99a	^39^
pHM1701	*lacI*^q^, greA D41A E44Y under control of Ptrc on pTrc99a, Ap^R^	Cashel’s lab collection
pHM1885	*lacI*^q^, greA-NTD (stop codon inserted after codon 75) under control of Ptrc on pTrc99a, Ap^R^	Cashel’s lab collection
pHM1887	*lacI*^q^, greA-CTD (residues 4–75 deleted) under control of Ptrc on pTrc99a, Ap^R^	Cashel’s lab collection
pHM1883	pGB2, pSC101 origin, Ptrc expression vector, Spec^R^	^19^
pHM1873	pSC101 origin, greA under control of Ptrc on pGB2, Spec^R^	^19^
pHM1854	pSC101 origin, greA D41A E44Y under control of Ptrc on pGB2, Spec^R^	^19^
pBbA2k	p15A origin, RFP under a P*tet* promoter, Km^R^	^59^
pBb-greA-His	*greA-his* under P*tet* promoter on pBbA2k, Km^R^	This study

### Error-prone PCR and mutant library construction

Random mutations in the *greA* open reading frame were generated by error-prone PCR as previously described^52^. Taq polymerase (New England Biolabs), which lacks proofreading activity, was used to PCR amplify the *greA* gene using the standard Taq buffer (10 mM Tris–HCl pH 9.0, 50 mM KCl, 1.5 mM MgCl_2_) supplemented with 0.1% Triton X-100 and 0.2 mg/ml BSA. The primers G11 and G6 (Supplementary Table S1) were used. During the first 10 amplification cycles, a slow decrease from denaturing to annealing temperature was applied (0.1 °C/s). The following 15 cycles were characterised by having a standard transition between denaturing and annealing.

### β-Galactosidase activity determination

β-Galactosidase activity measurements were performed as previously described^53^. Data are mean values from duplicate determinations in at least three independent experiments plotted with standard errors.

### GreA-His production and purification

To produce a C-terminal His-tagged GreA, the *greA* gene without the stop codon was PCR amplified using the primers greA_BglII and greA_BamHI (Supplementary Table S1). The resulting DNA fragment was cloned into pBbA2K using *Bgl*II–*Bam*HI restriction sites, resulting in the pBbA2k-GreA plasmid. A PCR fragment containing the sequence for a flexible peptide linker (Supplementary Table S1) made of 5 repeats of the motif (GlyGlyGlySer) was amplified with primers Linker_*Bgl*II and Linker_Histag_*Xho*I (Supplementary Table S1). The PCR fragment was digested with *Bgl*II and *Xho*I and ligated with *Bam*HI and *Xho*I cut plasmid pBbA2k-GreA with compatible cohesive ends. The resulting plasmid, pBb-greA-His, codes for a GreA variant containing a Gly-Ser linker and a His-tag.

His-tagged GreA variants were purified using Dynabeads His-Tag Isolation and Pulldown magnetic beads from ThermoFisher. Briefly, cells from 5 ml cultures (grown as described in the text) were collected by centrifugation and resuspended in 0.7 ml of binding/wash buffer (50 mM sodium-phosphate pH 8.0, 300 mM NaCl and 0.01% Tween-20) and sonicated (5 pulses of 20 s at 50% amplitude). After centrifugation, 20 µl of the magnetic beads were added to the supernatant, incubated for 5 min at room temperature, beads were washed 4 times with binding/wash buffer and proteins were eluted with 100 µl elution Buffer (300 mM imidazole, 50 mM sodium-phosphate pH 8.0, 300 mM NaCl and 0.01% Tween-20).

### Protein solubility test

Cells harbouring the different pGreA variant plasmids were grown overnight and 100 µl samples were taken. Separation of the soluble and insoluble fractions was performed as previously described^54^. GreA levels were determined by Western Blot.

### Gel electrophoresis and western blotting

LDS Sample Buffer (4 ×) with reducing agent from ThermoFisher was added to the samples before running on a NuPAGE 10% Bis–Tris Gel in MES SDS Running Buffer from ThermoFisher. Proteins were transferred onto a nitrocellulose membrane with an iBlot gel transfer system. His-tagged proteins were detected with an anti-His peroxidase conjugated antibody (1/500 in PBS-T with 2.5% of milk). GreA and RpoA were detected with mouse anti-GreA antibody (1/5000) and mouse anti-RpoA antibody (1/1000) in PBS-T with 2% of milk and then developed with peroxidase conjugated anti-mouse antibody. The signal was detected with a chemiluminescent reaction, using ECL™ Western Blotting from GE Healthcare, and detected by Molecular Imager ChemiDoc XRS System from BioRad.

To detect total protein in whole cell extracts, cells from 1 ml culture were centrifuged and 1 × LDS sample buffer added to the cell pellet sufficient to give an OD_600_ of 2.5. Samples were boiled, resolved on NuPAGE gels and then the gels were incubated for 30 min with a staining dye (0.5‰ Coomassie Brilliant Blue R-250, 10% Acetic Acid, 25% isopropanol) at room temperature. Next, the gels were rinsed with 10% acetic acid to eliminate the dye excess.

## Supplementary information

Supplementary Information.
