# Supplementary material for: Mutational analysis of Escherichia coli GreA protein reveals new functional activity independent of antipause and lethal when overexpressed
Source: Sci Rep. 2020 Sep 30;10:16074. doi: 10.1038/s41598-020-73069-1 (PMC7527559; doi:10.1038/s41598-020-73069-1)
Supplement: Supplementary file 1 — Supplementary Information. [file 41598_2020_73069_MOESM1_ESM.pdf]

Mutational analysis of *Escherichia coli* GreA protein reveals new functional activity independent of antipause and lethal when overexpressed.

Llorenç Fernández-Coll[1,3]*, Katarzyna Potrykus[2], Michael Cashel[1], Carlos Balsalobre[3]

Supplementary information

Figure S1: Overexpression of GreA from pTrc inducible plasmid. S-2

Figure S2: Viability of cells overexpressing GreA, GreA NTD or CTD. S-3

Figure S3: Expression of *fliC* distal (+1210) *lacZ* fusions. S-4

Figure S4: Solubility of the different GreA proteins. S-5

Figure S5: Basal GreA levels in Δ*dksA* Δ*greA* host cells carrying pGreA derived plasmids. S-6

Figure S6: Control experiments for the co-purification of GreA and RNAP. S-7

Figure S7: GreA binding to the RNA polymerase. Full western blots from Figure 5a. S-8

Table S1: List of primers used in this report S-9

References S-10

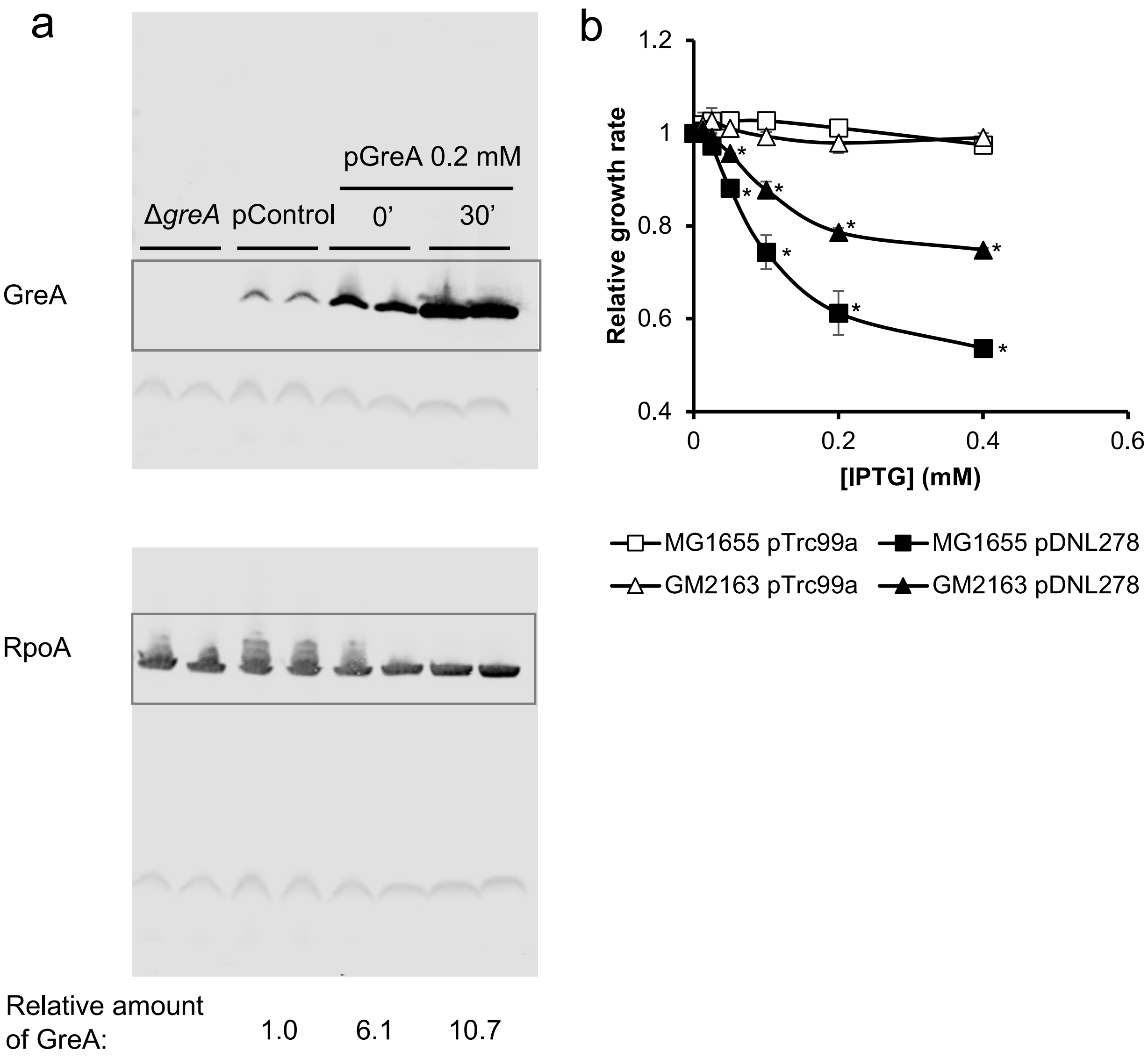

Figure S1: Overexpression of GreA from pTrc inducible plasmid. (a) Western Blot using monoclonal antibody against GreA and RpoA of MG1655 ΔgreA and MG1655 containing the plasmids pTrc99a (pControl) and pDNL278 (pGreA). Cells were grown in LB to up to similar densities and IPTG (0.2 mM) was added for 30 minutes. Cells from 1 ml culture were centrifuged and 1X LDS sample buffer added to the cell pellet sufficient to give an OD_{600nm} of 2.5. The amount of GreA is shown relative to MG1655 pControl, normalized to the amount of RpoA. (b) Strain MG1655 and GM2163 (*lacY1*) carrying pTrc99a (pControl) or pDNL278 (pGreA) plasmid was growth on LB with increasing concentrations of IPTG. Growth rate are shown relative to the growth rate of the culture without IPTG. Error bars represent SD from 3 independent cultures. The * stands for a pValue < 0.05.

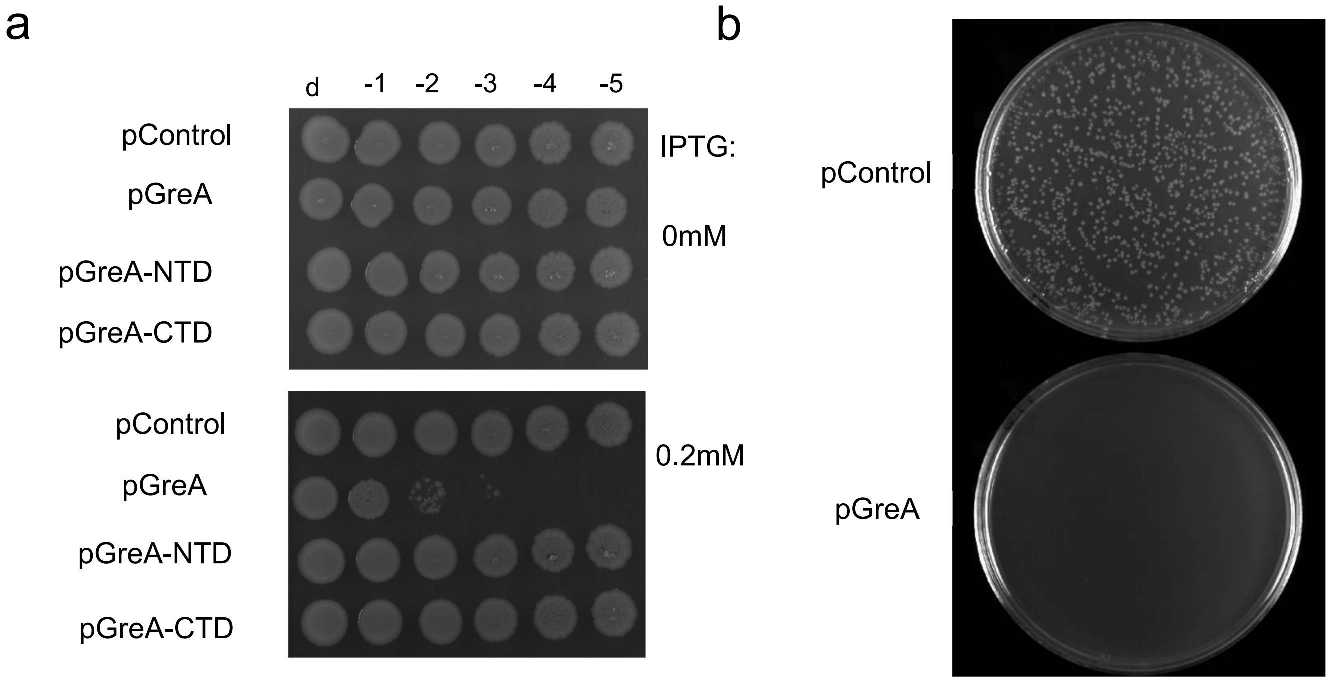

Figure S2: Viability of cells overexpressing GreA, GreA NTD or CTD. (a) MG1655 (WT) harbouring plasmid pTrc99a (pControl), pDNL278 (pGreA), pHM1885 (pGreA-NTD) and pHM1887 (pGreA-CTD) were grown in LB to similar densities and then serially diluted, with 2 µl of each dilution applied to LB plates containing 0 or 0.2 mM IPTG. (b) The strain MG1655 carrying pTrc99a (pControl) or pDNL278 (pGreA) plasmid was growth on LB agar plates with IPTG 0.2 mM. Under overexpression conditions, there are no visible colonies.

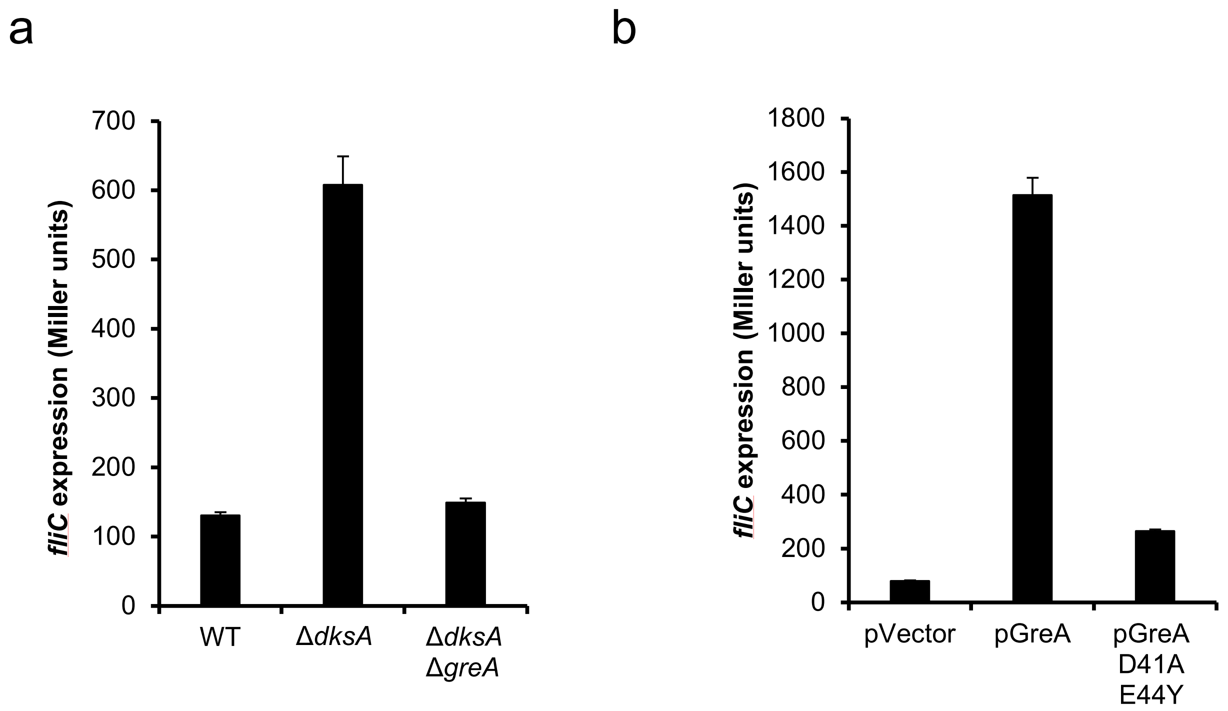

Figure S3: Expression of *fliC* distal (+1210) *lacZ* fusions. (a) The strains PRG16 (AAG1 *fliC::lacZ*) and its derivative mutant strains for *dksA* (PRG17) and for *dksA greA* (PRG18), were grown in LB at 37ºC up to an OD_{600nm} of 1.5 and β-galactosidase activity was determined, as previously shown in (1). (b) Expression of *fliC* in cultures of PRG18 (*dksA greA fliC*::*lacZ*) carrying the plasmids pHM1883 (pVector), pHM1873 (pGreA) and pHM1854 (pGreA D41A E44Y). Cultures were grown in LB at 37ºC up to an OD_{600nm} of 1.5. At OD_{600nm} of 0.1 the cultures were induced with 0.1 mM of IPTG. Average and standard deviation of β-galactosidase activity determination from three independent cultures are shown.

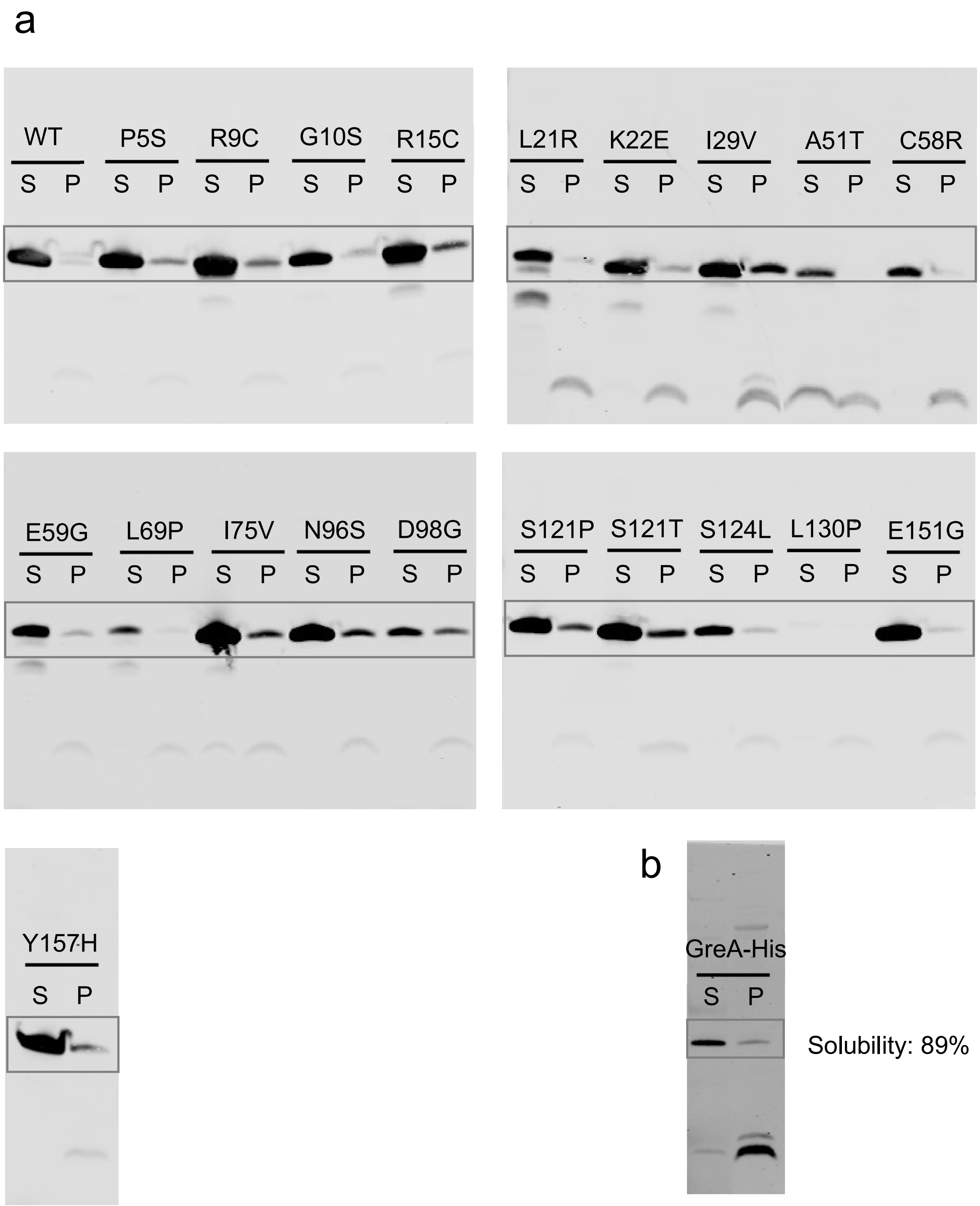

Figure S4: Solubility of the different GreA proteins. (a) Western Blot using monoclonal antibody against GreA of the soluble (S) and insoluble (P) fractions, from cultures of PRG18 (AAG1 *fliC::lacZ* (+1210) Δ*dksA* Δ*greA*) bearing plasmids pDNL278 (pGreA) or pTrc-GreA^{Mut}. Solubility %, are shown in table 1. (b) Western Blot using monoclonal antibody against GreA of the soluble (S) and insoluble (P) fractions, from cultures of the CF14758 strain (Δ*greA* Δ*greB*) containing pBb-GreA-his.

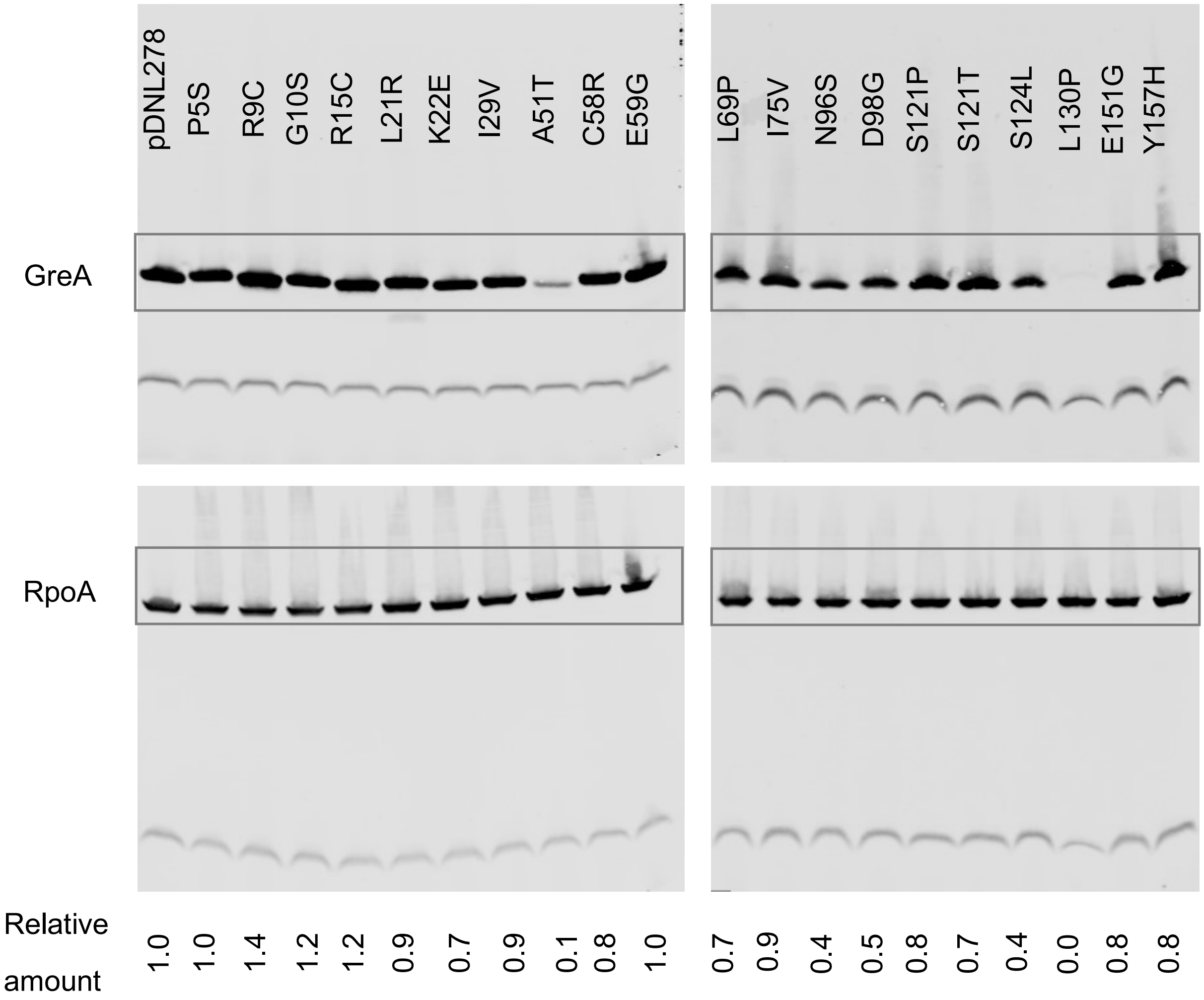

Figure S5: Basal GreA levels in Δ*dksA* Δ*greA* host cells carrying pGreA derived plasmids. Western Blot using monoclonal antibody against GreA and RpoA of cultures of PRG18 (AAG1 *fliC::lacZ* (+1210) Δ*dksA* Δ*greA*) bearing plasmids pDNL278 (pGreA) or pTrc-GreA^{Mut}. Cultures were grown under the same conditions as in figure 3 (LB at 37ºC up to an OD_{600nm} 1.5) and the soluble fraction was run in a SDS gel. The amount of GreA is normalized to the amount of RpoA.

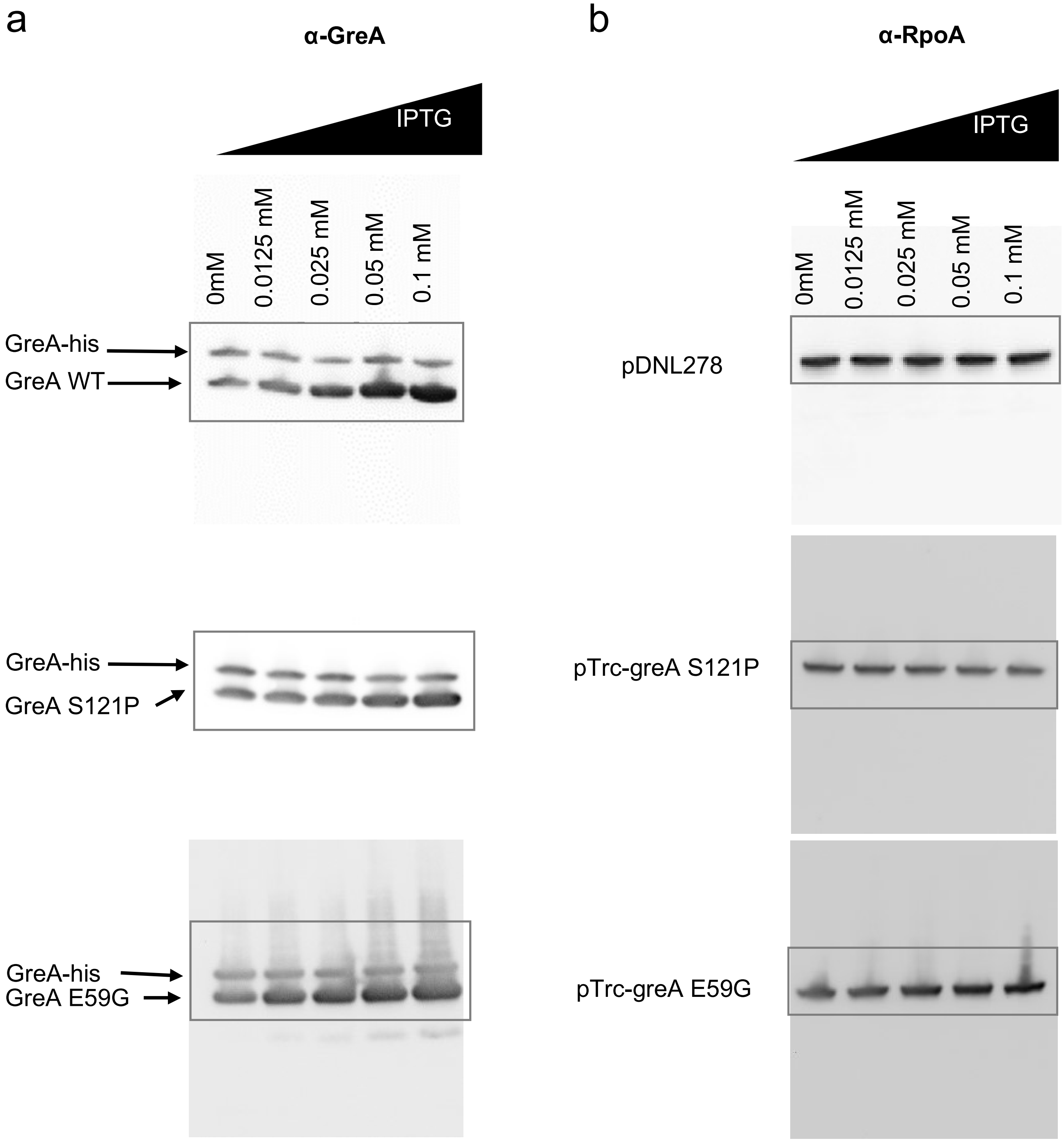

Figure S6: Controls for the co-purification of GreA and RNAP. Western Blot using monoclonal antibody against (a) GreA or (b) RpoA of cultures of CF14758 (Δ*greA* Δ*greB*) pBb-greA-His and pDNL278 (pGreA), E59G or S121P grown in LB with increasing amounts of IPTG. Cells from 1 ml culture were centrifuged and 1X LDS sample buffer was added to the cell pellet sufficient to give an OD_{600nm} of 2.5. (a) an increase in the amount of GreA from the pTrc plasmid is observed, while GreA-his remains constant. (b) No differences are observed in the amount of RpoA due to GreA overexpression.

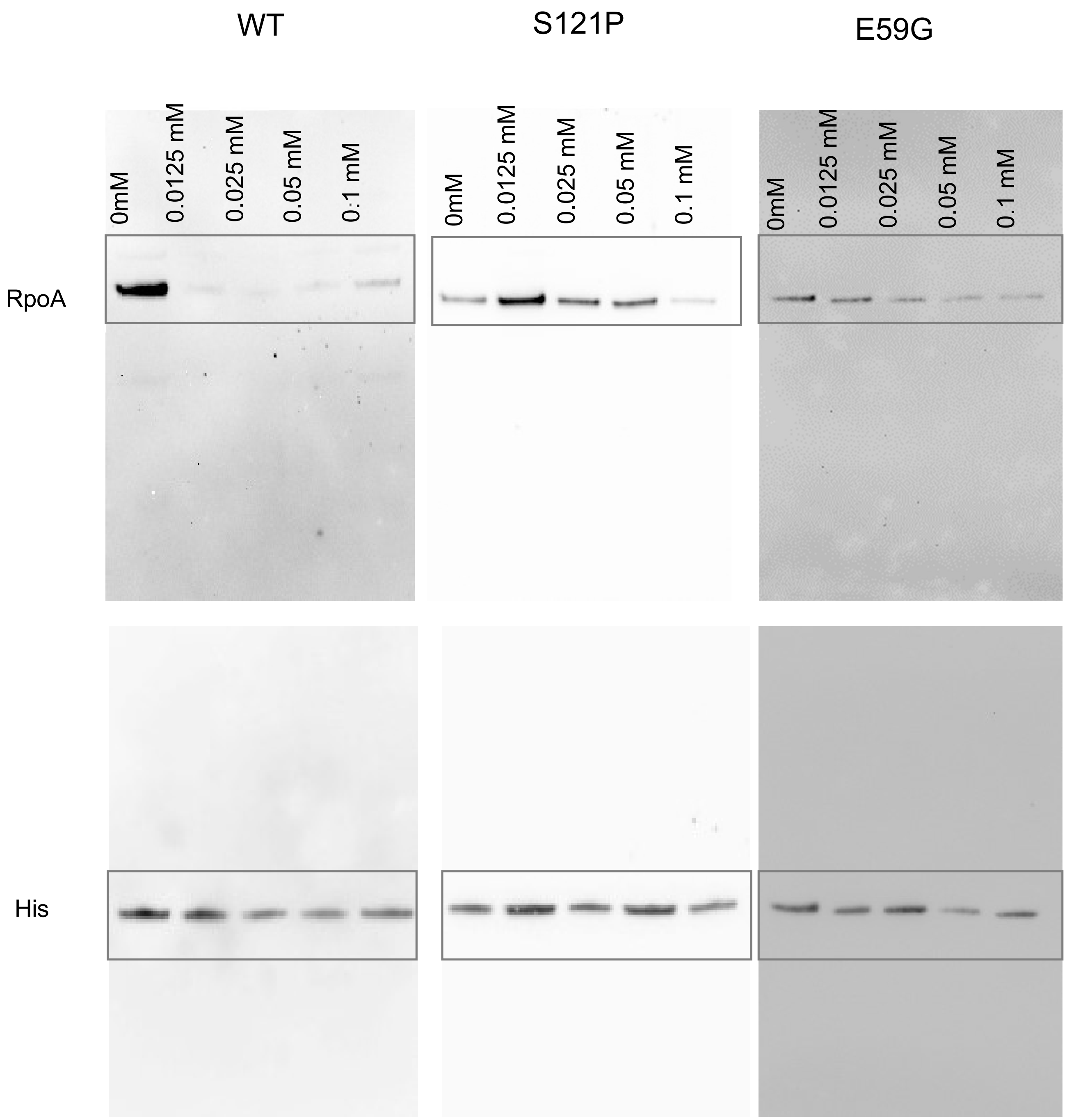

Figure S7: GreA binding to the RNA polymerase. Full western blots from Figure 5a.

Name	Sequence (from 5’ → 3’)
G11	cactgcagcaacatcttgagtattggg
G6	cagaattcatgcaagctattccgatgac
greA_BglII	gtagatctatgcaagctattccgatg
greA_BamHI2	gtcggatccttacaggtattccacctt
greA_BamHI	gtcggatcccaggtattccaccttaat
Linker	ggtggaggatcgggtggcggctcaggagggggctccggcgggggatctggggggggatcg
Linker_BglII	gtagatctggtggaggatcgggt
Linker_Histag_XhoI	actgctcgagttaatggtggtgatggtgatgcgatccccccccaga

Table S1: List of primers used in this report.

References

1. Aberg, A., Fernández-Vázquez, J., Cabrer-Panes, J. D., Sánchez, A., and Balsalobre, C. (2009) Similar and divergent effects of ppGpp and DksA deficiencies on transcription in Escherichia coli. *J. Bacteriol.* **191**, 3226–36
